# Hepatic VLDL secretion: DGAT1 determines particle size but not particle number, which can be supported entirely by DGAT2

**DOI:** 10.1194/jlr.M089300

**Published:** 2018-11-05

**Authors:** Zehra Irshad, Nikola Chmel, Raghu Adya, Victor A. Zammit

**Affiliations:** Translational and Experimental Medicine,* Warwick Medical School, Coventry CV4 7AL, United Kingdom; Department of Chemistry,† University of Warwick, Coventry CV4 7AL, United Kingdom

**Keywords:** triglycerides, lipoproteins, liver metabolism, lipids and cholesterol, metabolism, cardiovascular disease, metabolic syndrome, diacylglycerol acyltransferase 1, very low density lipoprotein

## Abstract

We investigated whether, in view of its activity being expressed on both aspects of the endoplasmic reticulum (ER; dual membrane topology), diacylglycerol acyltransferase 1 (DGAT1) plays a distinctive role in determining the triglyceride (TAG) content of VLDL particles secreted by the liver. Mice in which the DGAT1 gene was specifically ablated in hepatocytes (DGAT1-LKO mice) had the same number of VLDL particles (apoB concentration) in the plasma 1 h after Triton 1339 treatment, but these particles were approximately half the size of VLDL particles secreted by control mice and had a proportionately decreased content of TAG, with normal cholesterol and cholesteryl ester contents. Analyses of purified microsomal fractions prepared from 16 h fasted control and DAGT1-LKO mice showed that the TAG/protein ratio in the ER was significantly lower in the latter. Electron micrographs of these livers showed that those from DGAT1-LKO mice did not show the increased lipid content of the smooth ER shown by control livers. The effects of DGAT1- and DGAT2-specific inhibitors on apoB secretion by HepG2 cells showed that DGAT1 is not indispensable for apoB secretion and demonstrated redundancy in the ability of the two enzymes to support apoB secretion. Therefore, our findings show that DGAT1 is essential for the complete lipidation and maturation of VLDL particles within the lumen of the ER, consistent with its dual topology within the ER membrane. In the mouse, DGAT2 can support apoB secretion (particle number) even when TAG availability for full VLDL lipidation is restricted in the absence of DGAT1.

Hepatic secretion of VLDLs is an important determinant of lipidemia (1–3). Variations can result from a combination of changes in the number of particles (each containing a single molecule of apoB, a large amphipathic protein) and the lipid content [especially triglyceride (TAG)] of each particle (4). The mechanisms by which VLDL particles are assembled in the lumen of the endoplasmic reticulum (ER) of hepatocytes is mostly well understood (3, 5), but there are still aspects that require further insight. TAG is synthesized within the ER membrane and originates between the two leaflets of the membrane (6). A minority of this TAG is incorporated into primordial lipoprotein particles by association with apoB during the protein’s cotranslational insertion through the ER membrane, resulting in lipid-poor apoB-containing nascent particles being directed toward the ER lumen (7).

The rest of the nascent TAG is incorporated into cytosolic lipid droplets (LDs), which bud from the ER and are surrounded by phospholipid monolayer partly derived from the cytosolic leaflet of the ER (6). A complex of proteins appears to link the LD to the ER, facilitating synthesis and transfer of TAG to the LD (8). Although it is possible that some non-apoB-associated TAG enters the ER lumen at the time of synthesis, it is generally accepted that it is the pool of TAG within the cytosolic LDs that is the major source (9) of TAG for the enlargement [through a second lipidation step (10)] of the primordial, lipid-poor lipoprotein particles into the secretion-competent fully lipidated VLDL particles that are formed at the junction between the ER and the Golgi prior to secretion (10). But, in order for the cytosolic LD-TAG to be incorporated into VLDL-TAG, it needs to be hydrolyzed (11) [mostly to diglyceride, diacylglycerol (DAG) (12, 13)] and resynthesized at a site within the ER membrane that directs it toward the lumen and results in the formation of the non-apoB-associated TAG-containing LD within the lumen (7, 14, 15). Compositional studies of the luminal LDs showed that they have a distinct proteome of their delimiting membrane from that of cytosolic LDs (16). The use of TAG from ER-luminal LDs to lipidate the nascent lipoprotein particles results in the formation of fully lipidated, large VLDL particles, which gain other lipid and protein components in the process (10).

Therefore, the pathway through which cytosolic TAG acts as a precursor for the second-step lipidation of VLDLs within the ER lumen is important, in that it determines the availability of TAG for enlargement of VLDLs and, consequently, the ultimate size of the VLDL particles secreted. Larger particles (termed VLDL1) that are secreted under conditions of increased hepatic lipogenesis, hyperglycemia, and hyperinsuinaemia [e.g., T2D (2)] may be more proatherogenic due to their enhanced susceptibility to lipolysis by lipoprotein lipase in peripheral vasculature; their increased residence time in the plasma results in more highly modified, atherogenic, small dense LDL (17). However, TAG is not an easily membrane-permeant molecular species, and in spite of the demonstration that CideB may facilitate LD interaction with the ER (18), the known requirement for cytosolic TAG hydrolysis prior to its utilization for lipidation of VLDLs (see above) argues against the use of TAG molecules being transferred intact across the ER bilayer membrane.

We previously described a mechanism for the effective transfer of glycerides from cytosolic TAG into the ER lumen. This involves the hydrolysis of TAG of cytosolic LDs primarily to DAG and monoglyceride (12, 13, 19) and the transfer of DAG [a highly membrane-permeant molecule (20)] across the ER membrane. This necessitates the resynthesis of TAG on the luminal aspect of the membrane (21). In addition to the presence in the ER of proteins that facilitate the provision of acyl-CoA substrates at the luminal aspect of the ER (22), central to this mechanism is the bimodal distribution on either aspect of the ER membrane of the activity of diacylglycerol acyltransferase 1 (DGAT1) activity (23). DGAT1 appears to belong to a group of oligomeric membrane proteins that have bimodal orientations within the membranes in which they reside (24). The cytosol-facing subpopulation of DGAT1 enzyme appears to catalyze primarily the esterification of DAG with preformed FAs derived either endogenously through lipolysis of cytosolic LD-TAG or from the circulation (23, 25), whereas the lumen-facing DGAT1 is proposed to be central to the formation of the apoB-independent ER luminal LD, whose content is used to lipidate the nascent lipoprotein particle to form VLDLs (15, 23). By contrast, the other protein with major DGAT activity, DGAT2, is distributed in locations (including the cytosolic LDs) with its active site exclusively orientated toward the cytosolic compartment (26, 27). It has also been shown to be specialized for the esterification of nascent DAG and de novo synthesized FAs in mouse liver (28), HepG2 cells (25), and mouse brown adipocytes (29). Similarly, DGAT2 inhibition does not affect esterification of external oleate to TAG in the heart (30).

Therefore, we hypothesized that mice lacking DGAT1 specifically in hepatocytes (DGAT1-LKO mice) would be unable to form non-apoB-associated ER luminal LDs; this would impair the ability of the liver to form normal-sized VLDL particles, as the absence of intraluminal TAG synthesis would diminish the availability of non-apoB-associated TAG for second-step lipidation of nascent lipoprotein particles. Therefore, in this study we have examined the effect of hepatocyte DGAT1 deficiency on VLDL size, and TAG and apoB content, and find that, as would be predicted from the proposed role for the bimodal distribution of DGAT1 activity across the ER membrane, TAG lipidation of VLDL particles is markedly diminished in DGAT1-LKO mice.

## MATERIALS AND METHODS

### Materials

A TAG assay kit was purchased from Cayman Chemicals (Ann Arbor, MI). The ApoB ELISA kit was from LifeSpan Bioscience (Nottingham, UK). The RNeasy Mini kit was bought from Qiagen (Manchester, UK). [1-^14^C]-oleoyl-CoA (specific activity 60 mCi/mmol) was purchased from PerkinElmer (Coventry, UK). A cholesterol assay kit, Triton WR1339, tetrahydrolipstatin (THL), DGAT1 inhibitor T863, and all primers were purchased from Sigma-Aldrich (Irvine, UK). DGAT2 inhibitor (iJ) was prepared by Tocris Bioscience (Abingdon, UK). Heparinized syringes were from Abaxis UK Ltd. (York, UK).

### Animals

All procedures were performed in compliance with the regulations of the local ethics committee of the University of Warwick and licensed under Home Office regulations in the United Kingdom. To generate DGAT1-LKO mice, *Alb*.*Cre* and *dgat1*.*lox* mice were purchased as single strains from Jackson Laboratories (through Charles River, Margate, UK) to establish a double-transgenic colony. Original crossing of *dgat1* (homozygous) and *Alb.Cre* (heterozygous) produced double-transgenic offspring (*dgat 1* heterozygotes/*Alb.Cre* heterozygotes and WT). Once a sufficient number of mice were of the right age, sex, and genotype, they were then crossed to produce *dgat1.lox* (homozygotes)/*Alb.cre* (heterozygote and WT). The *Alb.Cre* WT littermates were used as controls. Only male mice were used.

### Methods

#### Triglyceride assay.

TAGs were measured in plasma samples, whole liver extracts, and isolated microsomes obtained from fed or fasted mice using a TAG assay kit according to the supplier’s protocol. Blood was collected from mice by cardiac puncture using heparinized syringes and centrifuged at 1,000 *g* for 5 min to yield plasma, which was assayed for TAG. Samples of livers (∼350 mg) or microsomal pellets (20 mg) were homogenized on ice in the NP40 substitute assay reagent of the assay kit and centrifuged at 10,000 *g* for 10 min at 4°C. The supernatant was removed and used for TAG measurement.

#### Cholesterol assay.

Total cholesterol and cholesteryl esters were measured by a colorimetric method (570 nm) using a cholesterol quantification kit (Sigma) according to the supplier’s instructions. Blood was collected from mice by cardiac puncture using heparinized syringes and centrifuged at 1,000 *g* for 5 min to yield plasma, which was assayed for cholesterol assay.

#### ApoB measurement.

ApoB was measured in mouse plasma (1 h after injection of Triton WR1339) using the ApoB Elisa kit for mouse ApoB (LifeSpan Bioscience) according to the supplier’s instructions. ApoB secretion rates by HepG2 cells were quantified using the ELISA kit for human ApoB (Mabtech, 2Bscientifc, United Kingdom).

#### DGAT activity assays.

DGAT activity was measured in liver extracts by quantifying the incorporation of [1-^14^C]-oleoyl-CoA into [^14^C] TAG in the presence of the second substrate 1,2-dioleoylglycerol as described previously (23). Frozen liver samples (∼200 mg) were thawed and homogenized on ice in 250 mM sucrose, 50 mM Tris, and 1 mM EDTA, pH 7.2. Supernatant was collected after centrifugation at 1,000 *g* for 10 min at 4°C and incubated on ice for 30 min either with carrier DMSO or with the DGAT 1 inhibitor [T863; 10 µM final concentration in DMSO)], DGAT2 inhibitor (iJ; 50 µM in DMSO) or with both inhibitors. After this incubation period, the aliquots (50 µl) were assayed for DGAT activity. The final assay medium contained 50 μM [1-^14^C] oleoyl-CoA (2.2 × 104 dpm/nmol), 500 μM 1,2 dioleoylglycerol dissolved in ethanol (final concentration of ethanol 0.25%), 2.5 mg/ml BSA, and 0.6% DMSO in 125 mM Tris-HCl buffer (adjusted to pH 7.4 using KOH at 37°C) containing 10 mM MgCl_2_ and 250 mM sucrose. The reaction was terminated after 10 min using chloroform/methanol (2:1, v/v). The chloroform layer was dried under a stream of nitrogen gas and resolubilized in chloroform (200 μl); the entire chloroform fraction was loaded onto a TLC plate coated with Silica Gel 60; and the lipid radioactive products were separated using hexane/diethyl ether/formic acid (70:30:1, v/v/v) as the mobile phase. A triglyceride standard (tripalmitin, 10 nmol) was used to identify the position of the triglyceride band after lipid separation. Lipid bands were visualized using iodine vapor. The radioactivity of each band was quantified after scraping into scintillation vials and quantifying the ^14^C radioactivity using a scintillation counter.

#### Real-time PCR quantitation of mRNA expression.

Total RNA was extracted from livers using the RNeasy Mini Kit (Qiagen) according to the manufacturer’s instructions. cDNA was prepared using reverse transcription. Briefly, RNA (1 μg) was mixed with oligodT (1 µl) in a final volume of 12 µl of RNase-free water. Samples were heated at 70°C for 5 min before chilling on ice. Subsequently, 8 µl of mixture containing RNase inhibitor (10 U/µl), dNTPs (10 mM), 5× reaction buffer, Bioscript reverse transcriptase, and RNase-free water was added to each sample. Samples were heated at 40°C for 60 min, and the reaction was stopped by heating to 70°C for 10 min. cDNA formed was mixed with 180 µl of nuclease-free water, stored at −20°C, and thawed once before quantification. Reverse transcription assay (RT-PCR) was performed using SYBR green dye, and expression for all the samples (n ≥ 3) was calculated by using the DCt method, incorporating the efficiencies of each primer pair. The variances of input cDNA were normalized against the levels of two housekeeping genes, L19 and B-actin. Melting curve analysis confirmed amplification specificity. Sequences of the primers used for each gene are given in **Table 1**.

**TABLE 1.** Sequences of primers used for quantification of mRNA expression of different genes in mouse livers

Gene	Forward	Reverse
mDGAT1	TTCCGCCTCTGGGCATT	AGAATCGGCCCACAATCCA
mDGAT2	AGTGGCAATGCTATCATCATCGT	TCTTCTGGACCCATCGGCCCCAGGA
mFAS	TGCGGAAACTTCAGGAAATGT	AGAGACGTGTCACTCCTGGACTT
mACC1	TGTCCACCCAAGCATTTCTTC	CATCCAACACCAGTTCAGTATACGT
mGPAT4	TGCCAAATGGGAGGTTTAAG	GCCACCATTTCTTGGTCTGT
mGPAT1	GTCCTGCGCTATCATGTCCA	GGATTCCCTGCCTGTGTCTG
mSCD1	CATCGCCTGCTCTACCCTTT	GAACTGCGCTTGGAAACCTG
mActin B	TTGCTGACAGGATGCAGAAG	CCACCGATCCACACAGACTA
mL19	GGAAAAAGAAGGTCTGGTTGGA	TGATCTGCTGACGGGAGTTG

#### Measurement of VLDL particle size using dynamic light scattering.

Tyloxapol (Triton WR1339, 200 µl; 2.5% in 0.9% PBS, 10 g of body weight) was injected 1 h before blood sampling to increase the VLDL fraction. Mice were terminally anesthetized using pentobarbitone, and blood was collected in tubes containing 5 μl of a saline solution of THL (200 µM final concentration). Blood from up to three mice was pooled to obtain a sufficient amount of purified VLDL fraction. The pooled blood was centrifuged at 1,000 *g* for 5 min. The supernatant was collected, aliquots were overlaid with KBr solution (density 1.008), and the VLDL fraction was purified by differential centrifugation at 27,000 *g* for 14 h at 12°C. The surface fraction containing the VLDL fraction was collected (200 µl) and used for dynamic light scattering (DLS) measurements immediately, without dialysis. Samples of the KBr medium were used as controls. DLS data were obtained using a Malvern Zetasizer Nano S 633 nm (Malvern, UK). Measurements of the particle size of VLDL fractions were obtained using 1.0 cm path length disposable micro-UV cuvettes (Brand, Germany). Samples were equilibrated at 37°C for 40 min prior to measurements, to stabilize Brownian activity and ensure reliable size determination, before aliquots (50 μl) were added to cuvettes. Measurements were performed at 37°C with 300 s equilibration time. Automated instrument parameters were used. Each measurement was repeated a minimum of seven times. The sample data obtained were normalized for intensity; means and SEMs were calculated. Viscosity (0.6864 cP at 37°C) and refractive index (1.330 at 37°C) of KBr solution were used. The refractive index of 1.450 was used for lipoprotein fractions. To calibrate the size measurement using these parameters, sample solutions were spiked using a 100 ± 3 nm National Institute of Standards and Technology traceable size standard (Nanosphere^TM^, ThermoScientific).

#### HepG2 cell culture.

HepG2 cells were purchased from the JCRB cell bank. They were cultured in RPMI 1640 medium supplemented with 10% FBS, 1% penicillin/streptomycin, 10 mM HEPES, and 2 mM l-glutamine at 37°C. Cells were subcultured every 5–6 days, just prior to reaching about 80–90% confluency, into fresh vented culture flasks or into 6-well plates for experimental work. Unless specified otherwise, all experiments were performed in phenol-free medium with 10% FBS, 1% penicillin/streptomycin, 10 mM HEPES, 2 mM l-glutamine, and 0.75 mM oleate with 25% BSA. Cells treated with either iDGAT1 (T863; 10 µM) or iDGAT2 (iJ; 5 µM) and apoB secretion into the medium quantified after 4 h at 37°C using a human ApoB ELISA kit (see Materials). Initial experiments ascertained that secretion was linear up to this time point. ApoB levels were measured in the medium according to the manufacturer’s protocol. Levels were normalized to protein concentrations.

#### Preparation of microsomal fraction from mouse livers.

The livers were homogenized using a Teflon-glass homogenizer in 10 vol of ice-cold medium containing 250 mM sucrose, 1 mM EGTA, and 10 mM Tris (pH 7.2). The homogenates were centrifuged for 10 min at 1,000 *g*. The supernatant was centrifuged twice for 20 min at 10,000 *g*, and subsequently at 76,000 *g* for 4 h. The purified microsomal fraction was recovered from the high-speed pellet.

#### Electron microscopy.

Tissue sampling and fixation was performed as described in ref. 31. Images were obtained using a Jeol 2100Plus microscope equipped with a Gatan OneView Camera.

#### Statistical analyses.

Differences between means for independent groups of data were analyzed by Student’s *t*-test or paired Student’s *t*-test, as indicated. Nonparametric data analysis was performed using the Mann-Whitney *U* test.

## RESULTS

### Liver and plasma parameters of DGAT1-LKO mice

**Fig. 1.**
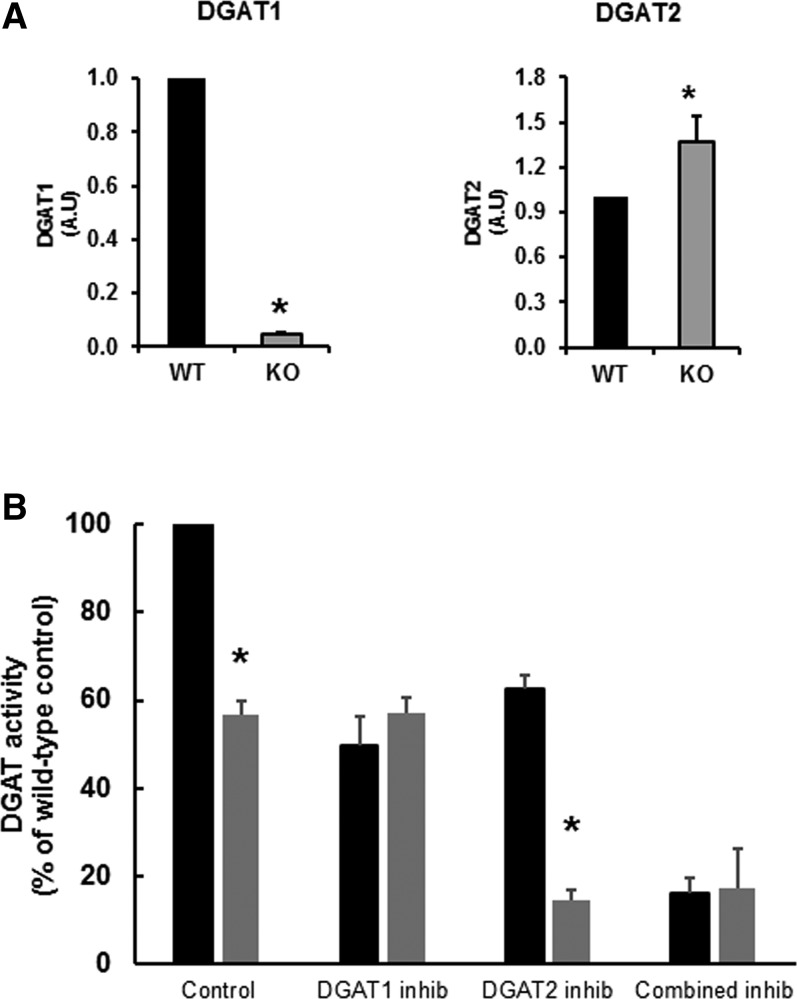
Effect of the conditional knockout of the *dgat*1 gene in hepatocytes (DGAT1-LKO) on mRNA expressions of the *dgat*1 and *dgat2* genes (A) and hepatic activity of DGAT in the presence of specific DGAT1 or DGAT2 inhibitors (inhib) (B). A: The loss of DGAT1 expression in the liver of DGAT1-LKO mice (KO) was verified. This was accompanied by a modest, but significant, increase in the mRNA expression of DGAT2. Values, in arbitrary units (A.U.), for each gene are expressed relative to the geomean of mRNA expression of two housekeeping genes, L19 and Actin B, for five separate liver RNA preparations. B: DGAT activities were measured in liver whole homogenates of frozen liver samples in the absence or presence of specific inhibitors of DGAT1 or DGAT2 or a combination of the two (see Methods). The control DGAT activity in liver of WT mice was 0.98 µmol/min/g liver at 37°C. *Statistically different values (*P* < 0.02).

The hepatocyte-specific loss of DGAT1 expression resulted in virtual elimination of the mRNA expression for the *dgat1* gene (**Fig. 1A**). There was a small increase in the mRNA expression for DGAT2, indicating that DGAT2 expression was not induced significantly to compensate for the absence of DGAT1. The loss of DGAT1 expression was accompanied by the loss of approximately half the DGAT activity in the liver (Fig. 1B). The difference in overall hepatic DGAT activity in DGAT1-LKO mice was comparable to the degree of inhibition of overall DGAT activity by a specific DGAT1 inhibitor (T863) (Fig. 1B). This was consistent with the observation that in DGAT1-LKO livers, the overall activity of DGAT was unaffected by T863, whereas it was markedly inhibited by compound iJ (29), which is a specific inhibitor of DGAT2. These observations confirmed that the *Cre-Lox* strategy (see Methods) used to specifically knock down *dgat1* gene expression in the hepatocytes of DGAT1-LKO mice had effectively resulted in the total absence of the functional enzyme from the hepatocytes. Minor residual DGAT activity in the livers of both WT and DGAT1-LKO mice after combined exposure to DGAT1 and DGAT2 inhibitors indicates that some DGAT activity is attributable to other enzymes able to esterify DAG to TAG in the in vitro assay and/or to DGAT1 activity in other cell types within the liver.

**Fig. 2.**
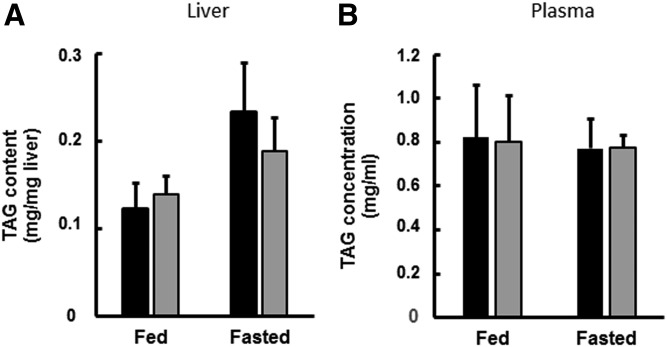
Triglyceride concentrations in liver and plasma of WT and DGAT1-LKO mice. WT (black bars) or DGAT1-LKO (gray bars) mice were used either in the fed state (l h into the 12 h light period) or in the fasted state, 6 h into the light period of a 12 h light/12 h dark diurnal cycle. Blood was obtained, using heparinized syringes, by cardiac puncture of anesthetized animals, and liver samples were obtained and frozen immediately. Plasma was prepared from the blood samples by centrifugation at 10,000 *g* for 5 min. Values for liver content (A) and plasma concentration (B) are means (± SEM) for five separate determinations on separate mice. The increase in liver TAG content after 6 h fasting did not reach statistical significance.

Neither the TAG content of the liver nor the plasma concentration of TAG were affected relative to WT control values in DGAT1-LKO mice in the fed state (**Fig. 2**). Although there was a tendency for TAG content of liver to increase after 6 h fasting, particularly in WT animals, the differences were not statistically significantly higher than the TAG content of fed control mice.

### Changes in hepatic lipogenic enzyme mRNA expression in DGAT1-LKO mice

**Fig. 3.**
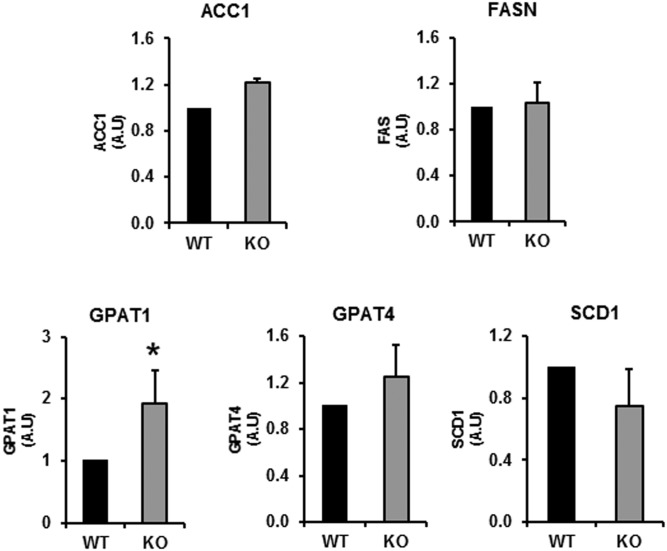
Expression of several lipogenic genes in the livers of WT and DGAT1-LKO mice. The levels of mRNA expression of several lipogenic enzymes were measured in the liver of WT and DGAT1-LKO mice. There was a significant increase in GPAT1 mRNA relative to WT controls. * *P* < 0.05. There were no changes in the mRNA expression of other lipogenic genes. Values, in arbitrary units (A.U.), for each gene are expressed relative to the geomean of mRNA expression of two housekeeping genes, L19 and actin B, for five separate liver RNA preparations. ACC1, acetyl-CoA carboxylase 1; GPAT, glycerolphosphate acyltransferase; SCD1, steroyl-CoA decarboxylase 1.

We quantified the mRNAs for several lipogenic genes in the livers of fed mice to ascertain whether the absence of DGAT1 from hepatocytes was likely to have altered the lipogenic potential of hepatocytes in DGAT1-LKO mice (**Fig. 3**). With one exception (GPAT1), none of the lipogenic genes tested showed any increase in mRNA expression. The 2-fold increase in GPAT1 mRNA may indicate that, in the absence of DGAT1, a SREBP1c-independent response (32) by the liver may occur to increase the esterification of newly synthesized FAs. GPAT1 is localized in the mitochondrial outer membrane (33), where it competes for acyl-CoA with carnitine palmitoyltransferase 1, thus attenuating the entry of FAs into the mitochondrial matrix for β-oxidation (32). Like DGAT2 (15, 25), GPAT1 is specialized for the esterification of de novo synthesized FAs (34), which are the preferred substrates for DGAT2 in mouse liver (28). Although of interest, a study of the implications of these changes for the partitioning of hepatic FAs between esterification and oxidation was outside the scope of the present study.

### VLDL composition in DGAT1-LKO mice

**Fig. 4.**
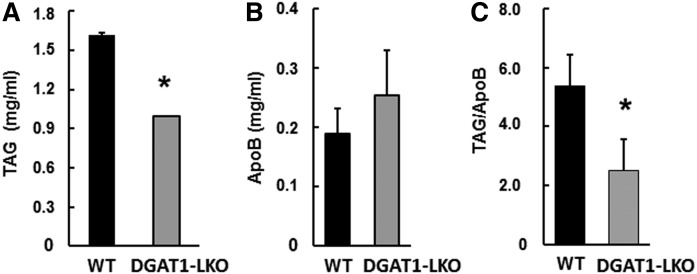
Plasma concentration of TAG and apoB in DGAT1-LKO mice and controls after a 16 h fast. Mice were fasted for 16 h and then injected intraperitoneally with Triton WR-1339 to inhibit lipoprotein lipase activity. After l h, they were anesthetized and bled; blood samples were collected using heparinized syringes, and the plasma fraction was obtained by centrifugation at 10,000 *g* for 5 min. The plasma samples were frozen, and the concentrations of TAG (A) and apoB (B) were measured as described in *Methods*. C: The TAG/apoB ratios were obtained for individual mice, and the ratio values were analyzed (paired *t*-test). Values are means (± SEM) for determinations on five separate mice for each condition. * *P* < 0.02 (values for DGAT1-LKO mice that are significantly different from those for WT controls).

The levels of VLDLs were raised by treatment of the mice with Triton-WR1339 (Tyloxapol) 1 h before blood sampling to enable more accurate analyses (see Methods). Initially, the choice of 6 h fasting was made to avoid the interference from chylomicron content of TAG and apoB, but also to prevent significant mobilization of FAs from adipose tissue, and a consequent increase in hepatic TAG content, which occurs with longer duration of fasting both in control and DGAT1-deficient mice (35). This minimized the possibility that the composition of VLDLs would be influenced directly by the TAG availability within the liver (9). The data in Fig. 2 show that the contents of TAG in liver and plasma were not different between control and DGAT1-LKO mice after 6 h fasting. However, after 16 h fasting, plasma TAG was 40% lower in DGAT1-LKO mice (**Fig. 4A**); by contrast, there was no significant change in the concentration of apoB in the plasma; indeed, there tended to be an increase (Fig. 4B). These combined effects resulted in a significant 55% decrease in the TAG/apoB ratio in the plasma of DGAT1-LKO mice after 16 h fasting (Fig. 4C). There were no changes in either cholesterol or cholesteryl ester concentrations in the plasma (not shown). The decrease in TAG/apoB ratio suggested that there was a major decrease in the size of individual VLDL particles. Therefore, we decided to test this inference directly.

### Sizing of VLDL particles

**Fig. 5.**
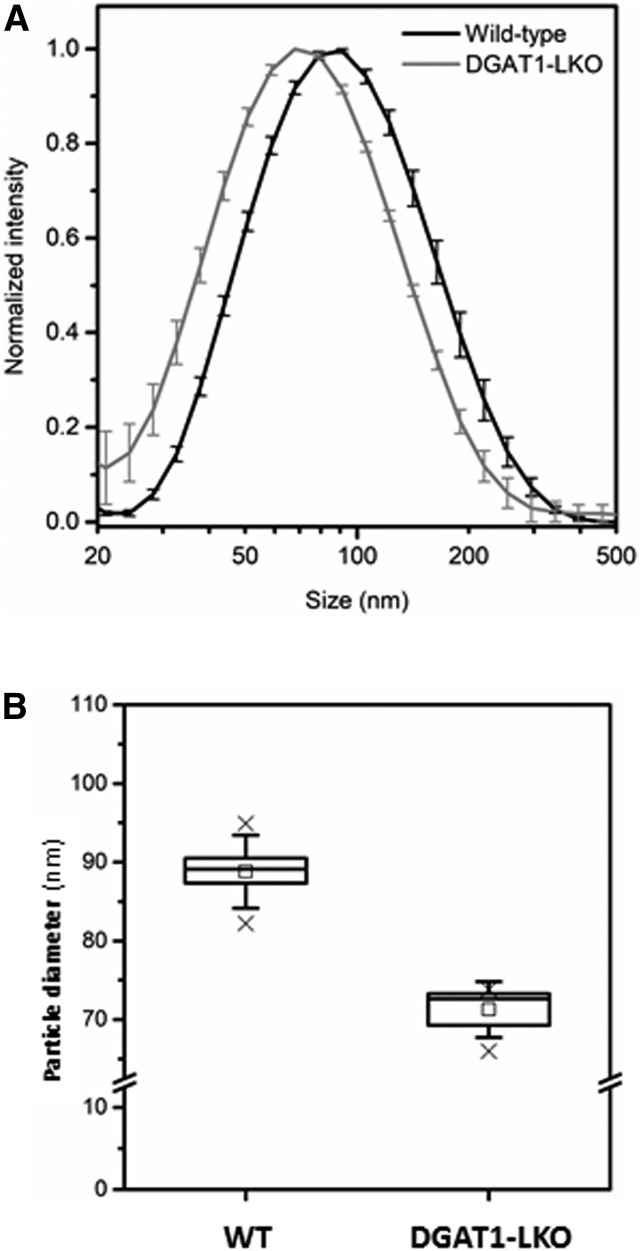
VLDL particles secreted by the liver of DGAT1-LKO mice are smaller than those secreted by WT animals. Plasma samples were prepared from either WT or DGAT1-LKO male mice fasted for 6 h, and the VLDL fraction (d < 1.006) was prepared by step-gradient centrifugation, as described in Methods. A: Particle size determination was performed using DLS. Values are means (± SEM) for five separate determinations and are statistically significantly different between DGAT1-LKO and WT (*P* < 0.01). B: The mean values are indicated by open squares and the upper and lower limits of the respective ranges by ×. Horizontal lines are medians; boxes show the interquartile range, and error bars show the SD.

Using DLS (see Methods), we measured directly the size of VLDL particles isolated from DGAT1-LKO and WT mice. **Figure 5** shows that there was a statistically significant decrease (20%) in the diameter of VLDL particles in DGAT1-LKO mice. Assuming that VLDL particles behave like spheres, this decrease in diameter is equivalent to a 45% decrease in particle volume, which is consistent with the magnitude of the decrease in TAG/apoB ratio observed above (Fig. 4C). Therefore, this provides further evidence that the absence of DGAT1 in hepatocytes results specifically in the secretion of smaller VLDLs by the liver.

### Effects of specific inhibition DGAT1 and DGAT2 on apoB secretion in HepG2 hepatocytes

**Fig. 6.**
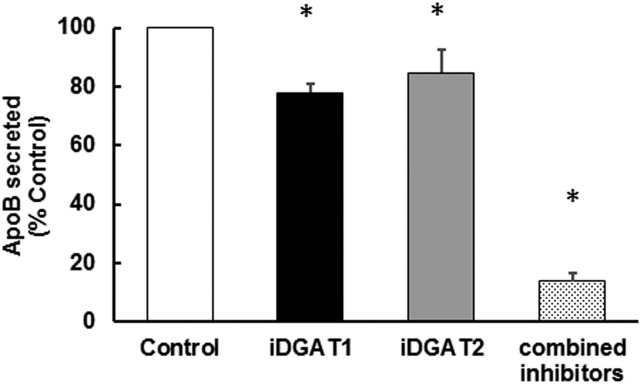
Incomplete redundancy in the ability of DGAT1 and DGAT2 to support ApoB secretion in HepG2 cells. HepG2 cells were incubated with oleic acid and glucose as substrates, as described in Methods. ApoB secretion into the medium was measured (using ELISA) for a period of 4 h in the absence (control) or presence of specific inhibitors of DGAT1 (iDGATl) and/or DGAT2 (iDGAT2) as indicated. The rate of ApoB secretion in control cells was 86.26 ng ApoB/h/mg cell protein. Values are expressed as a percentage of the rate observed for control cells in the same experiment for n = 3 separate determinations. Each apoB measurement was performed in duplicate. * *P* < 0.02 (values that are significantly different from control).

The above data indicated that the absence of DGAT1 from hepatocytes only affects the maturation and lipidation of the nascent lipoprotein particles, and not the number of particles secreted. Because the latter depends on the initial lipidation of apoB during cotranslational translocation of the protein across the ER membrane, this step can potentially be supported by cytosol-facing (overt) DGAT1 (15, 23, 25) and/or by DGAT2, the catalytic site of which is entirely cytosol-facing. Our previously published observations (25) showed that inhibition of DGAT1 affects primarily the utilization of preformed FAs for TAG synthesis and that, although DGAT2 also contributes substantially to this process, this enzyme is highly specialized for the incorporation of de novo synthesized FAs and nascent DAG into cellular and secreted (VLDL) TAG (15, 23, 25). However, in those experiments we did not measure apoB secretion. Therefore, in order to address the question of whether apoB secretion is affected when each of the two enzyme activities is inhibited in turn, we performed experiments with HepG2 cells incubated with oleic acid (to stimulate lipoprotein secretion) and with specific inhibitors of each of the two enzymes. The results in **Fig. 6** show that neither inhibition of DGAT1 nor DGAT2 individually markedly suppressed apoB secretion, although there were small decreases after inhibition of either enzyme. By contrast, ApoB secretion was almost totally inhibited when both enzymes were simultaneously inhibited (Fig. 6). This is consistent with the data (Fig. 4) indicating that, in vivo, even in the absence of DGAT1, DGAT2 by itself is able to support apoB secretion in smaller, TAG-poor particles.

### TAG content of the ER

**Fig. 7.**
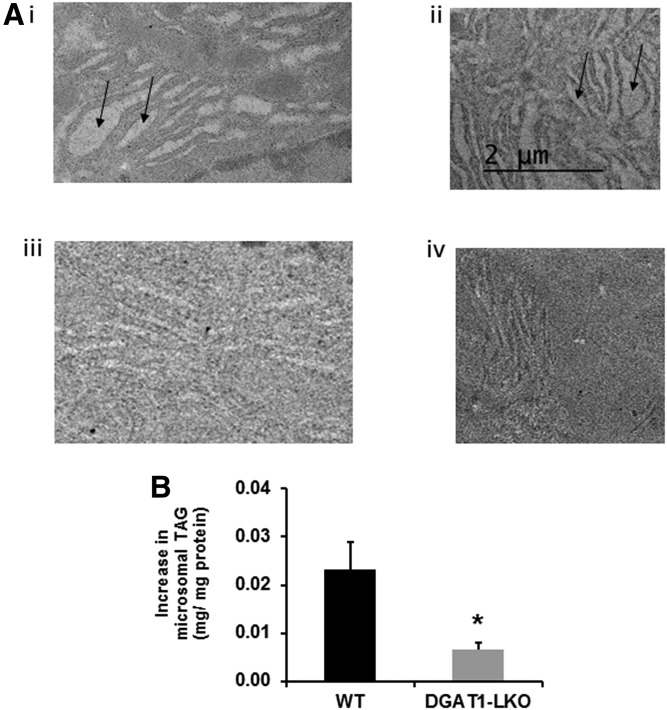
Difference in the TAG content of microsomal lumen between fasted control and DGAT1-LKO mouse liver. Mice were fasted for 16 h to increase the supply of nonesterified FAs to the liver. Total liver TAG increased to the same extent in control and DGAT1-LKO mice (6.0 ± 0.5 and 5.3 ± 0.3 mg TAG/per mg liver in control and DGAT1-LKO livers, respectively. A: Electron micrographs of liver sections obtained from two separate control mice (i, ii) and two DGAT1-LKO mice (iii, iv) show the difference in TAG content of smooth ER (arrows). B: The difference in the increase in TAG content of purified microsomal fractions obtained from control and DGAT1-LKO mice is indicated. * *P* < 0.05.

In an attempt to demonstrate directly that deficiency of DGAT1 from hepatocytes in vivo interferes with the formation of ER TAG, we obtained electron micrographs of liver sections from control and DGAT1-LKO mice (**Fig. 7**). We also prepared purified microsomal fraction preparations and measured their TAG and protein content. When fed animals were used, no discernible differences were observed between control and DGAT1-LKO mice with either technique (not shown). However, when FA availability to the livers was increased by fasting the animals for 16 h prior to sacrifice, we observed regions of smooth ER in control animals which were replete with TAG (Fig. 7A i, ii). These TAG-filled membranes were not observed in sections obtained from DGAT1-LKO mouse livers (Fig. 7A iii, iv) in which the ER looked very similar to that in fed animals. Similarly, whereas upon 16 h fasting there was an increase in the TAG/protein ratio in purified endoplasmic reticular fractions of 23.00 ± 0.01 ng TAG/mg microsomal protein in control mouse microsomes, the increase in microsomes from fasted DGAT1-LKO mice was only 7.10 ± 0.01 ng TAG/mg protein (Fig. 7B). Consequently, the increase in TAG/protein ratio was significantly lower (*P* < 0.05) in microsomes from DGAT1-LKO mice than in control mice.

## DISCUSSION

The main observation of this study is that the absence of DGAT1 from hepatocytes results in a markedly decreased diameter of VLDL particles, equivalent to the halving of their volume. This was accompanied by a decrease the TAG/apoB ratio of VLDL particles. These changes were achieved notwithstanding a similar overall content of TAG in the liver. This has implications for the understanding of the respective functions of DGAT1 and DGAT2 in the assembly of mature VLDL particles secreted by the liver. The two enzymes catalyze the same, final reaction of TAG synthesis, are coexpressed in hepatocytes and other cell types, and are partially redundant for the esterification of preformed FAs to TAG. However, DGAT2 has one specialized nonredundant function: in HepG2 cells (25), murine hepatocytes and mouse liver in vivo (28), and in mouse brown adipocytes (29), DGAT2 is specialized for the synthesis of TAG from nascent DAG (containing newly synthesized FAs) and de novo synthesized FAs (15). This is consistent with observations that DGAT2 associates with lipogenic enzymes (e.g., GPAT, Fpat1, and SCD1) in various systems (15), and its expression is commonly induced together with that of lipogenic genes under the control of SREBP1c and/or ChREB (36, 37). In the liver (15, 25) and brown adipocytes (29), DGAT2 also contributes to the esterification of preformed FAs that arise either endogenously through lipolysis or are derived from the circulation (either of dietary or adipose origins) to DAG, in hepatocytes and, to a greater extent, in brown adipocytes. However, it is DGAT1 [which is more active in the liver (23, 25)] that appears primarily to catalyze the reesterification of DAG and monoacylglycerol generated by TAG hydrolysis. It is noteworthy that in white adipocytes (white adipose tissue), DGAT1 has the exclusive function of catalyzing the reesterification of FAs generated by endogenous TAG lipolysis (38). Similarly, although DGAT2 mRNA expression is relatively high in cardiomyocytes, its specific inhibition does not affect esterification of oleate exogenously supplied to the heart (30). These examples provide evidence for specialized roles of DGAT1 and DGAT2 in different cell types (15). Previous studies have quantified the effects of specific knockdown or overexpression of either DGAT1 or DGAT2 in liver and have shown that DGAT2 activity is primarily associated with the development of hepatic steatosis (39–41), whereas overexpression of DGAT1 specifically increases TAG secretion by the liver, and increased lipid accumulation in the smooth ER (31).

Rapid cycling between lipolysis and reesterification is a characteristic of hepatocyte TAG metabolism; in an earlier study we estimated that the rate of TAG-DAG cycling is more than an order of magnitude higher than the rate of FA utilization for TAG secretion in primary rat hepatocytes (12). DGAT1 is the primary contributor to this, but DGAT2, which is partially localized to the cytosolic LDs (27) also contributes to it (25). The function of this extensive hydrolysis-reesterification cycling may be related to the requirement to control the net rate of lipolysis of cytosolic LD-TAG to DAG, which is the immediate source of ER luminal non-apoB-associated TAG used to lipidate the nascent lipoprotein particles to a secretion-competent size (15). VLDL-TAG secretion is related to hepatic TAG content (mostly within cytosolic LD) (9), and inhibition of lipolysis of this TAG pool results in a decrease in VLDL-TAG secretion (11).

A role for DAG in the effective transfer of the glyceride moiety across the ER membrane has been established (15) and is made possible when DAG is diverted away from reesterification on the cytosolic aspect of the membrane, crosses the ER membrane, and is reesterified on the ER luminal aspect. Previous studies (21, 23, 42–44) showed that a substantial proportion of DGAT1 activity of the hepatocyte ER is not accessible to the cytosolic long-chain acyl-CoA pool and can catalyze DAG esterification only if acyl-CoA is made accessible to the luminal aspect of the ER membrane. This trans-membrane system for synthesis of a pool of TAG that gives rise to non-apoB-associated TAG within the ER lumen has been reconstituted in vitro using ER vesicles (22) and is made possible by the dual topology of DGAT1 within the ER membrane (23). This is consistent with the previous observations that, in the absence of DGAT1, steatosis from preformed FAs is much diminished (35), presumably because this would require the activity of overt (cytosol-facing) DGAT1.

The current observations are also consistent with the proposed exclusive role of latent DGAT1 activity in the formation of a pool of ER lumen TAG that is used for second-step lipidation of nascent VLDLs. Thus, in DGAT1-LKO mice, the TAG content of the VLDL particles secreted by the liver is reduced approximately by half (Fig. 4) with a corresponding halving of the volume of the lipoprotein particles (Fig. 5), as would be anticipated from a requirement for ER lumen-facing (latent) DGAT1 activity for the formation of TAG in apoB-free LDs in the ER lumen. The difference between VLDL size in WT and DGAT1-LKO mice was specifically associated with the TAG content of the particles, as cholesterol and cholesteryl ester contents were unaffected. It is noteworthy that the size and TAG content of chylomicrons secreted by the intestine is also decreased in the absence of DGAT1 from enterocytes (45), in which the relationship between cytosolic TAG and TAG-rich lipoprotein secretion appears to be similar to that in the liver (46).

The observation that plasma apoB concentrations after 1 h Tyloxapol treatment was unaltered in DGAT1-LKO mice compared with control mice indicates that DGAT2 activity is largely sufficient, by itself, to meet the TAG synthesis requirements for the assembly of nascent (TAG-poor) lipoprotein particles. This suggests that TAG synthesized by DGAT2 plays a central role in determining apoB secretion (i.e., particle number). Conversely, DGAT2 alone cannot maintain the size and TAG content of secreted VLDLs (this study), although, as expected from its role in esterifying de novo synthesized FAs, previous studies have shown that it is fully capable of supporting hepatic steatosis (cytosolic LD-TAG synthesis) induced by an LXRα agonist, i.e., through endogenous hepatic lipogenesis (35). In HepG2 cells, both DGAT1 and DGAT2 inhibitors decreased apoB secretion but only moderately; these decreases were small compared with that observed when both activities were inhibited simultaneously.

As DGAT2 is largely responsible for the esterification of de novo FAs to nascent DAG (25, 28, 29), these observations are consistent with the central importance of FA synthesis for hepatic secretion of TAG. This obligatory link between FA synthesis and VLDL-TAG secretion is a long-established observation (47, 48). Therefore, the ability of DGAT2 to maintain particle number secretion but not full VLDL lipidation and maturation is consistent with previous observations that, although de novo synthesized FAs constitute a small proportion of total TAG secreted (49–51), their incorporation is essential for TAG secretion (47, 48). Their contribution toward secreted VLDL-TAG is markedly increased upon a high-carbohydrate (lipogenic) diet, when de novo FA synthesis is enhanced (51). Fig. 7 presents evidence that the TAG content of the ER is increased to a much greater extent in control than in DGAT1-LKO mice when exogenous FA supply is increased.

**Fig. 8.**
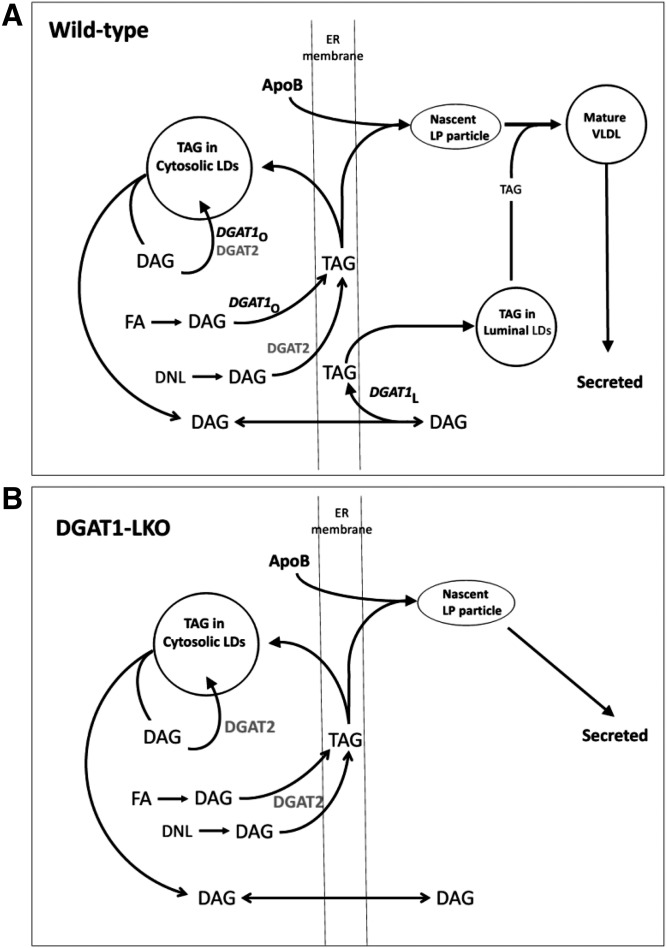
Proposed roles of DGAT1 and DGAT2 in the synthesis and secretion of VLDL TAG in WT (A) and DGAT1-LKO (B) mouse liver, based on the experimental observations. In WT mouse hepatocytes, both overt DGAT1 *(DGAT1*o) and DGAT2 contribute to the (re)esterification of DAG with preformed (exogenous) FAs, although DGATlo plays the major role. Conversely, DGAT2 is specialized for the esterification of de novo synthesized FAs (DNL) and nascent DAG. The TAG formed within the ER membrane is either used for the formation of cytosolic LDs or is associated with apoB during its cotranslational insertion through the ER membrane to form nascent lipoprotein particles. DAG equilibrates through the membrane to become the substrate for latent DGAT1 (*DGAT1* L), which forms TAG destined for ER luminal LDs, and lipidation of nascent particles to form mature, TAG-rich VLDL, which is secreted. B: DGAT1 is absent (DGAT1-LKO), and no TAG enrichment (enlargement) of the nascent lipoprotein particles occurs on the luminal aspect of the ER membrane. DGAT2-catalyzed TAG synthesis on the cytosolic aspect of the membrane is sufficient to sustain apoB secretion.

A graphical representation of the roles of DGAT1 and DGAT2 in the assembly of VLDL particles in WT and DGAT1-LKO mice, based on the current findings, is presented in **Fig. 8**.

The size of secreted VLDL, which primarily reflects their TAG content, is an important index of the pathogenicity of the dyslipidemia associated with the (pre)diabetic state (52, 53). VLDL1-type particles are larger and more TAG-rich, are increasingly secreted in diabetes (52, 53), and are more atherogenic (53, 54). Therefore, elucidation of the roles of DGAT1 and DGAT2 in the mechanism whereby the enlargement, through further lipidation of nascent VLDL particles with TAG, prior to their secretion is achieved is important, although it is appreciated that observations on DGAT2 in mice cannot invariably be extrapolated to humans (55). Our studies show that DGAT1 is the enzyme responsible for determining VLDL particle size, whereas DGAT2 may be more important for determining the number of VLDL particles secreted, as it is capable of maintaining apoB secretion in the absence of DGAT1.
